# Diagnostic Performance of Elastography Compared With Cytopathology in the Evaluation of Thyroid Nodules Suspicious for Malignancy

**DOI:** 10.7759/cureus.107538

**Published:** 2026-04-22

**Authors:** Manuela Restrepo Gomez, Tatiana Arroyave Peña, Alexander Aguilar, Sebastian Peláez, Diana Marin, Carlos González Vásquez, Ana María Posada

**Affiliations:** 1 Department of Diagnostic Radiology, Universidad Pontificia Bolivariana-Cedimed, Medellín, COL; 2 Department of Radiology, Universidad Pontificia Bolivariana-Cedimed, Medellín, COL; 3 Department of Radiology, Cedimed, Medellín, COL; 4 Department of Medical Sciences, Universidad Pontificia Bolivariana, Medellín, COL; 5 Department of Radiology, Universidad Pontificia Bolivariana, Medellín, COL; 6 Department of Radiology, Cedimed, Hospital Pablo Tobón Uribe, Medellín, COL

**Keywords:** biopsy, elastography, neoplasms, thyroid nodule, ultrasound

## Abstract

Introduction: Cytopathologic evaluation is the gold standard for the diagnosis of thyroid cancer. Ultrasound elastography is a noninvasive technique that assesses tissue stiffness and may help select patients for biopsy based on the stiffness of thyroid nodules.

Methods: A cross-sectional observational study was conducted in patients with thyroid nodules suspicious of malignancy who underwent qualitative and semiquantitative elastography. These results were compared with the cytopathologic findings of each lesion following fine-needle aspiration biopsy. Bethesda categories V and VI were considered malignant. Bethesda III and IV nodules, corresponding to indeterminate and follicular categories, were not included in the analysis as malignant nodules.

Results: A total of 106 thyroid nodules were evaluated, predominantly in females, with a mean age of 54 years. Most nodules were benign (83.1%). Concordance between qualitative elastography and malignancy was practically null, with a Kappa value of -0.0503 (95% CI: -0.10 to -0.004). The median semiquantitative value was similar across all Bethesda categories: 1.14 (0.79-2) in benign nodules (Bethesda II), 1.59 (0.69-4.12) in Bethesda V nodules, and 1.68 (0.36-3.92) in Bethesda VI nodules. Elastography did not allow clear discrimination between benign and malignant nodules using a precise numerical threshold. A cutoff value of 1.48 is proposed, as it yielded the best balance between sensitivity (64.29%, 95% CI: 38.76-86.66) and specificity (63.77%, 95% CI: 51.98-74.1).

Conclusion: Elastography demonstrated limited utility in differentiating benign from malignant nodules in this study. Further research is needed to define its clinical role in the implementation of noninvasive approaches for the early detection of thyroid cancer.

## Introduction

According to data from the American Cancer Society, there were an estimated 2,290 deaths from thyroid cancer in 2025, with the disease being three times more common in females [1]. In Colombia, thyroid cancer ranks as the fifth most common cancer, with an incidence of 9.4 per 100,000 females and a mortality rate of 3.5, according to the National Cancer Institute [2].

Non-palpable thyroid nodules are common incidental findings in the general population, with a prevalence ranging from 19% to 67%, and most patients remain asymptomatic. However, the global incidence of thyroid cancer has been rising, and thus, ultrasound has become essential in evaluating the morphological characteristics of nodules to assess malignancy risk [3,4]. Although the majority of nodules are benign, up to 7-15% may represent malignant lesions [5], underscoring the importance of accurate evaluation.

Ultrasound elastography (UE) is a noninvasive technique that evaluates tissue stiffness in thyroid nodules, while fine-needle aspiration cytology (FNAC) remains the reference standard for the diagnosis of malignancy in suspicious thyroid nodules. The diagnostic performance of UE compared to cytopathology has been the subject of numerous studies and recent systematic reviews. In general, UE demonstrates sensitivity for malignancy ranging from 76% to 90% and specificity between 66% and 87% in thyroid nodules, depending on the technique used (strain, shear wave, strain ratio elastography) and the study population [6-8].

In recent years, elastography has been evaluated in numerous studies regarding its utility as a complement to conventional ultrasound and the TI-RADS (Thyroid Imaging Reporting and Data System) [9] classification system for predicting malignancy risk in thyroid nodules. As an adjunct to ultrasound, elastography could improve diagnostic accuracy in the evaluation of suspicious nodules and reduce reliance on biopsy. Sun et al. report in their meta-analysis a sensitivity of 71% and specificity of 76% for differentiating benign from malignant nodules using elastography, while Nell et al. report sensitivity up to 85% and specificity of 80% [10,11]. However, some studies have demonstrated the contrary, showing low concordance in distinguishing benign from malignant nodules compared to biopsy [2,3].

To date, no studies in Colombia have compared the diagnostic performance of elastography versus histopathology for the detection of thyroid cancer in an outpatient setting. This study evaluates the utility of elastography in identifying malignant thyroid nodules and its comparison with fine-needle aspiration biopsy, aiming to contribute to improved diagnostic accuracy and early detection of thyroid cancer in the Colombian population.

## Materials and methods

Study design

A cross-sectional observational study was conducted in 2024 to evaluate the diagnostic performance of thyroid elastography in determining malignancy risk in thyroid nodules, in comparison with fine-needle aspiration biopsy, with the aim of improving early detection of thyroid cancer.

Participants

Patients were consecutively enrolled at a high-complexity diagnostic medical center in Medellín, Colombia. All participants had thyroid nodules detected on prior ultrasound studies and had been referred for fine-needle aspiration biopsy between August 2023 and December 2024, and attended the diagnostic center to undergo the procedure.

Patients were included in the study if they were older than 18 years with thyroid nodules detected on prior ultrasound classified as “high risk” according to TI-RADS, who had been referred for fine-needle aspiration. TI-RADS is a standardized classification system that uses ultrasound characteristics of nodules to assess malignancy risk [9]. Patients classified as “high risk” were defined as those with TI-RADS 3 (≥2.5 cm), TI-RADS 4 (≥1.5 cm), or TI-RADS 5 (>1 cm) nodules. Only patients with a medical referral who provided informed consent for biopsy, elastography, and study participation were enrolled.

Patients were excluded if they presented with active or chronic local skin or soft tissue infection near the evaluation site, had high bleeding risk (due to anticoagulant therapy or blood dyscrasias), or if they obtained a cytopathology result of Bethesda I, which is equivalent to an unsatisfactory specimen.

Once participants were identified and enrolled, biopsy and elastography were performed consecutively during the same clinical consult. The radiologist performing the elastography was blinded to the prior TI-RADS classification and cytopathology results, and the pathologist analyzing the specimen was unaware of the elastography findings and TI-RADS category. Variables collected for each patient included age, sex, personal or family history of thyroid cancer, and prior thyroid biopsies. For each patient, the number of nodules studied and the location of each were recorded. The TI-RADS classification at the time of referral and a new TI-RADS classification assigned by the radiologist performing the biopsy were both documented for each nodule. In addition to TI-RADS, qualitative and semiquantitative elastography results were obtained and recorded prior to the biopsy.

Study methods

The index test was thyroid elastography, performed using a Toshiba APLIO 400 ultrasound system (Toshiba Corporation, Tokyo, Japan) with a high-frequency transducer (15 MHz). The examination was performed by one of the three radiologists with prior training in elastography. No interobserver calibration was performed due to logistical and scheduling constraints among the staff and the medical center.

Elastography is a diagnostic imaging modality that produces elastograms generated by transducer pressure applied to the tissue, or by the effect of the patient's own physiological movements, such as cardiac pulsation, respiration, or muscle contraction, on the structure of interest. The repetitive compression applied to the skin deforms the underlying tissues, and upon release of pressure, the specific elasticity of the target tissue can be measured, in this case, the thyroid nodule at risk for malignancy. Accordingly, minimal and repetitive compression was applied over the thyroid nodule selected for biopsy until the equipment confirmed that adequate pressure had been achieved for elasticity measurement.

**Figure 1:**
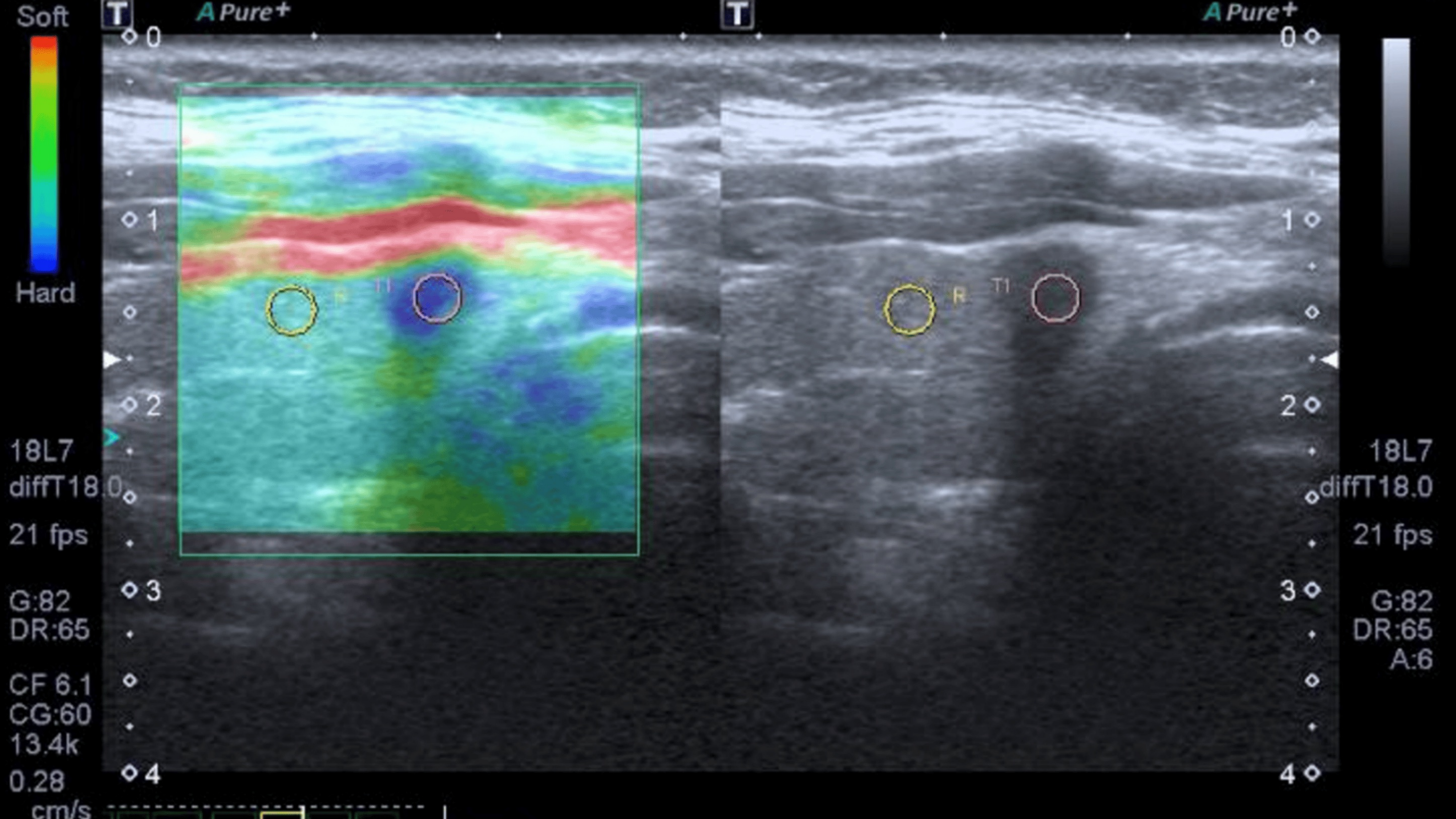
Ultrasound elastography. A 48-year-old male patient with a right thyroid nodule classified as ACR TI-RADS 5. Quantitative elastography score was 5, and qualitative elastography A score was 4. The pathology report was suspicious for papillary carcinoma, Bethesda category V. ACR TI-RADS: American College of Radiology Thyroid Imaging Reporting and Data Systems.

During the examination, areas of calcification and cystic regions were avoided, as these alter the elasticity of the region of interest. Representative images were acquired from multiple sites within the nodule using a region of interest (ROI), with five to 10 measurements obtained to calculate the mean stiffness value (Figure 1).

Qualitative results were interpreted using the Asteria Score (AS) scale: A score 1 indicates a soft nodule with elasticity equal to adjacent tissue; A score 2 indicates an elastic, predominantly deformable nodule; A score 3 indicates a predominantly rigid nodule, less deformable than adjacent tissue; and A score 4 indicates a rigid, non-deformable nodule. The semiquantitative elastography result was interpreted using the Strain Index in kilopascals (numerical scale from 1 to 10) and was documented in a standardized database [6,12,13].

Following conventional ultrasound and elastography, ultrasound-guided fine-needle aspiration biopsy was performed on the suspicious nodule or nodules. The procedure was carried out using aseptic technique with a 22-26G gauge needle. Under direct ultrasound guidance, a puncture was performed through the skin into the target nodule, and cells were obtained by aspiration or capillary action. Specimens were preserved on slides and sent to the pathology laboratory for analysis, where the pathologist assigned a classification according to the Bethesda System [14]. Samples were obtained from the most sonographically suspicious areas and from the zone of greatest stiffness identified on elastography, while maintaining the pathologist's blinding to elastography results.

Finally, elastography results were compared with the reference standard, which was the cytopathology result of each nodule according to the Bethesda classification [14]. This system categorizes the cytopathologic results of thyroid nodules evaluated by fine-needle aspiration from category I through VI based on malignancy characteristics. Bethesda I denotes a non-diagnostic or unsatisfactory specimen; Bethesda II includes benign nodules; Bethesda III is assigned to atypia of undetermined significance or follicular lesions of undetermined significance; Bethesda IV encompasses follicular neoplasms or suspicious follicular neoplasms; Bethesda V includes lesions suspicious for malignancy; and Bethesda VI is reserved for malignant nodules.

For this study, nodules classified by qualitative elastography as an A score of 3 or 4 on the AS scale were considered malignant. For the quantitative scale, different cutoff values were analyzed with their corresponding sensitivity, specificity, and predictive values to determine whether a threshold could be identified to indicate malignancy. These were compared against malignant nodules as determined by cytopathology (Bethesda V and VI).

Data were collected systematically using a standardized form. Each patient was assigned a numeric code, and all identifying information was removed to minimize bias and ensure data privacy and confidentiality. Analysis was performed once the cytopathology result was received and the variable form for each patient was fully completed.

Statistical analysis

Patient characteristics were described as mean (standard deviation, SD) or median (interquartile range, IQR) according to the assumption of normality, with other variables reported as absolute values and percentages (%).

The nodule was the unit of analysis for estimating malignancy frequency. Nodules positive for malignancy by cytopathology (Bethesda V or VI) were placed in the numerator, and all Bethesda II nodules classified as negative for malignancy were placed in the denominator. Overall and sex-stratified malignancy frequencies (%) were reported with 95% confidence intervals (95% CI). The prevalence ratio (PR) of malignancy by sex was also calculated.

For thyroid nodules, the initial American College of Radiology (ACR) TI-RADS classification at referral was compared with the new ACR TI-RADS classification assigned by the radiologist at the time of biopsy, with concordance estimated using the Kappa index (95% CI). Semiquantitative and qualitative elastography results were then compared with overall histopathology using the Kruskal-Wallis test, and with grouped results (malignant vs. benign) using the Mann-Whitney U test to determine association. Malignancy prevalence was calculated for each category of nodule characteristics.

For the assessment of concordance between elastography and histopathologic results, qualitative elastography was classified as negative (A score 1 or 2) or positive (A score 3 or 4), and the Kappa statistic was estimated excluding indeterminate and follicular nodules (Bethesda III and IV). Additionally, receiver operating characteristic (ROC) curves were used to explore possible semiquantitative elastography cutoff values to assess their diagnostic performance for determining malignancy (Bethesda V, Bethesda VI). Analyses were performed using SPSS 29.0 (IBM Corp., Armonk, NY) and Jamovi 2.4. A two-tailed p-value of 0.05 was considered statistically significant. Bethesda III and IV were excluded from the final analysis.

## Results

Of 106 patients referred for thyroid nodule biopsy, four obtained a non-diagnostic Bethesda I result. Among the remaining 102 patients, the mean age was 54.3 years, and 87 (85.3%) were women. Nine out of 10 patients had no personal or family history of thyroid cancer (Table 1). Among those who had undergone prior thyroid biopsies, one (6.3%) had an unknown prior cytopathology result, and the remaining 15 (93.7%) had a Bethesda result of III or lower, indicating non-malignant nodules.

**Table 1:** Sociodemographic characteristics of patients who underwent elastography at a reference diagnostic center in Medellín (n = 102). SD: standard deviation; Min-Max: minimum – maximum; N/A: not applicable.

Variable		n	%	Mean, SD (Min-Max)
Age, years	All	N/A	N/A	54.3 (13-86)
Sex	Female	87	85.30	N/A
	Male	15	14.70	N/A
Personal history of thyroid cancer	Yes	2	2	N/A
	No	100	98	N/A
Family history of thyroid cancer	Yes	7	6.9	N/A
	No	95	93.1	N/A
Prior thyroid biopsies	Yes	16	15.7	N/A
	No	86	84.3	N/A

Some patients presented with more than one nodule. Accordingly, the TI-RADS concordance analyses correspond to 106 nodules. The majority were in the right thyroid lobe (101, 95.3%), and of these, more than half (57, 56.6%) were in the inferior pole. Following the procedure, nodules were most frequently classified as TI-RADS 3 (n = 36, 34.0%) and TI-RADS 4 (n = 43, 40.6%).

In most cases, the ACR TI-RADS classifications obtained prior to the procedure were concordant with those assigned by the radiologist performing the biopsy (Kappa: 0.65; 95% CI: 0.53-0.76). Most discordant cases occurred because the prior classification was higher than that assigned by the study radiologist. Of the 29 TI-RADS 3 nodules, five were reclassified to a lower category, and only one to TI-RADS 4. Of the 53 TI-RADS 4 nodules, 14 received a lower reclassification, and of the 24 TI-RADS 5 nodules, five were reclassified to a lower category.

**Figure 2:**
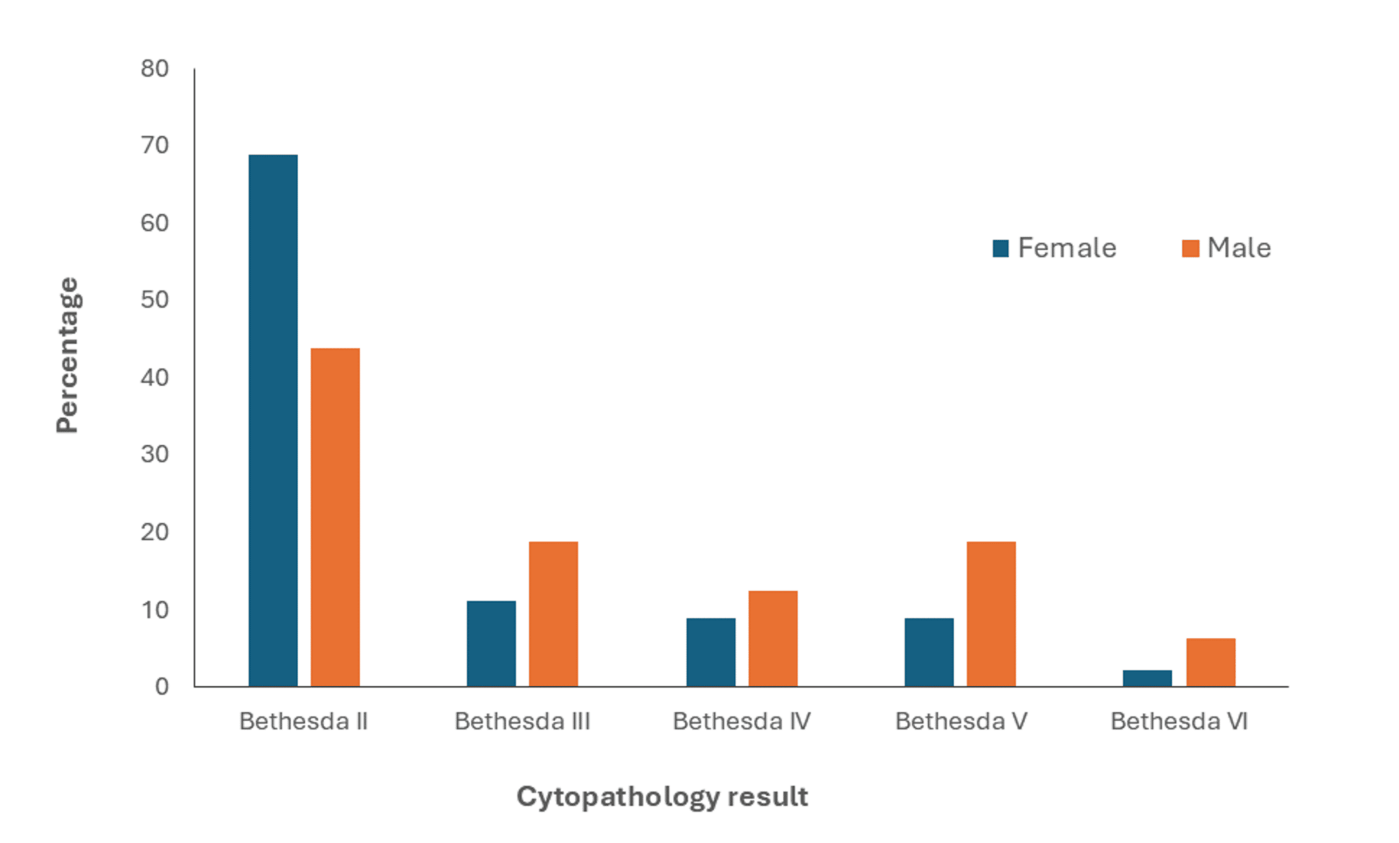
Cytopathology results by sex. Benign nodules (Bethesda II), indeterminate nodules (Bethesda III and IV), and malignant nodules (Bethesda V and VI).

Of 106 nodules submitted for cytopathology, 13 (12.2%) received a Bethesda III and 10 (9.4%) a Bethesda IV classification. Of the remaining 83 nodules, most were benign (69, 83.1%), classified as Bethesda II, while 14 nodules (16.9%) yielded malignant cytopathology (Bethesda V and VI) (Figure 2). Malignant cytology was found in four (25%) men and 10 (11.1%) women (PR: 2.62; 95% CI: 0.51-13.4).

**Figure 3:**
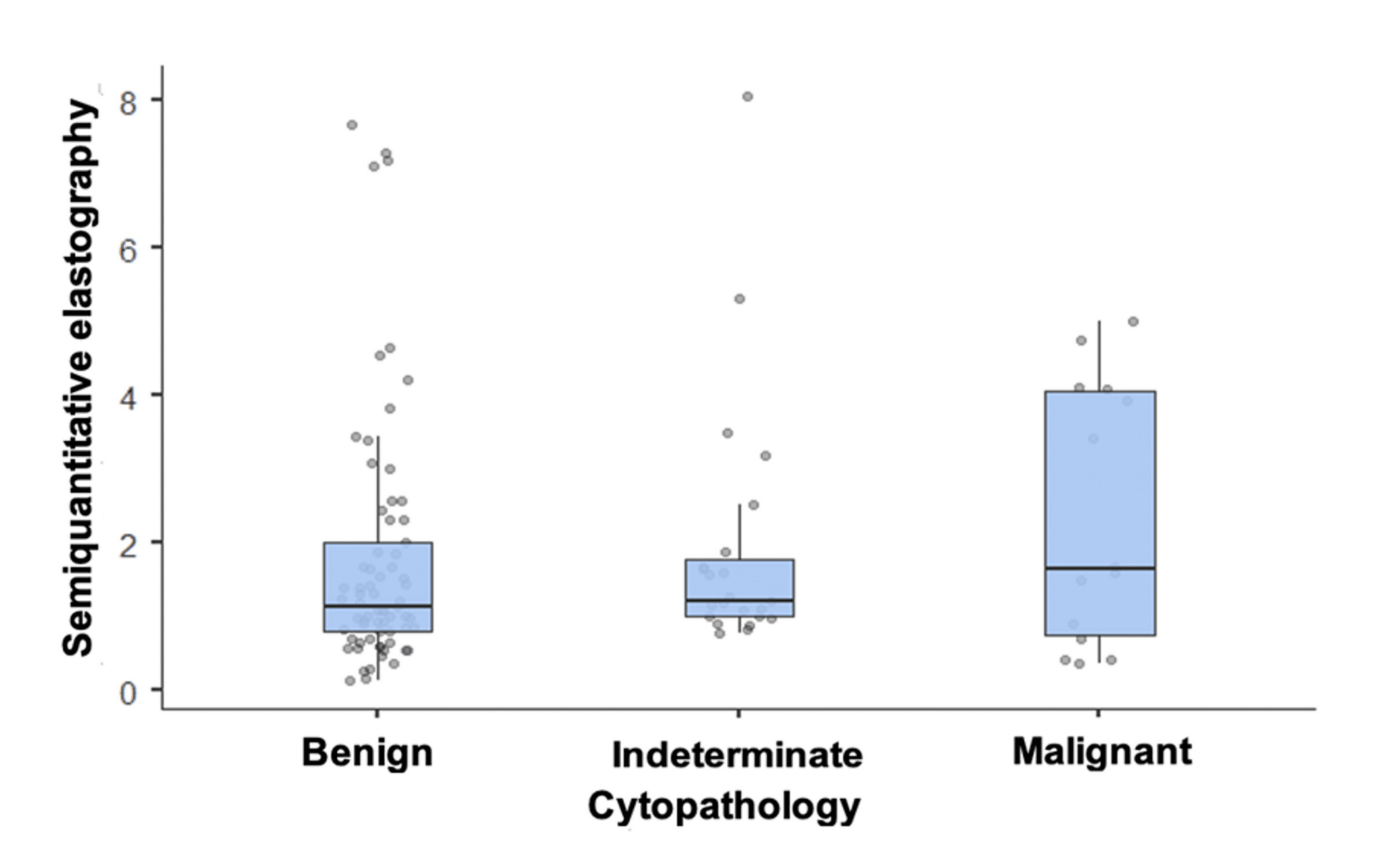
Semiquantitative elastography results according to malignancy as determined by cytopathology.

Regarding the application of elastography to thyroid nodules, analysis of semiquantitative elastography values revealed that the median semiquantitative values were similar across all cytopathology categories (p-value: 0.396), falling below 2 in all groups, without allowing discrimination between benign and malignant nodules (Table 2 and Figure 3).

**Table 2:** Semiquantitative elastography results in relation to cytopathology findings.

Cytopathology	Minimum–maximum	Mean (SD)	Median (P25–P75)
Bethesda II	0.14 - 7.69	1.781 (1.729)	1.14 (0.79 - 2)
Bethesda III	0.78 - 3.19	1.363 (0.631)	1.15 (1.01 - 1.57)
Bethesda IV	0.82 - 8.07	2.656 (2.373)	1.62 (0.97 - 3.5)
Bethesda V	0.42 - 5.0	2.444 (1.835)	1.59 (0.69 - 4.12)
Bethesda VI	0.36 - 3.92	1.987 (1.8)	1.68 (0.36 - 3.92)
Total	0.14 - 8.07	1.887 (1.725)	1.2 (0.85 - 2.32)

The most frequent qualitative elastography result was an A score of 2 (54 nodules, 50.9%). Although concordance between qualitative elastography and cytopathology was practically null (Kappa: -0.0503; 95% CI: -0.10 to -0.004), the closest association was observed in benign nodules (Bethesda II), which more frequently had qualitative values below 2 (48 nodules, 69.5%). Among nodules with an A score of 3 or 4, 21 nodules (56.7%) corresponded to histopathologically benign results, while nine nodules (4.3%) corresponded to malignant nodules. Among the indeterminate nodules (Bethesda III and IV), 16 (23.2%) had an A score of 1 or 2, and seven (18.9%) had an A score of 3 or 4 (Table 3).

**Table 3:** Cytopathology results expressed as Bethesda categories in relation to qualitative elastography results (A score). Values in blue represent concordant results, with Bethesda V and VI and A score 3 and 4 considered malignant. Kappa: -0.0503 (95% CI: -0.10, -0.004).

Qualitative elastography	Cytopathology					Total
	Bethesda II	Bethesda III	Bethesda IV	Bethesda V	Bethesda VI	
A score 1	11	1	3	0	0	15
A score 2	37	9	3	5	0	54
A score 3	16	3	3	1	1	24
A score 4	5	0	1	5	2	13
Total	69	13	10	11	3	106

**Figure 4:**
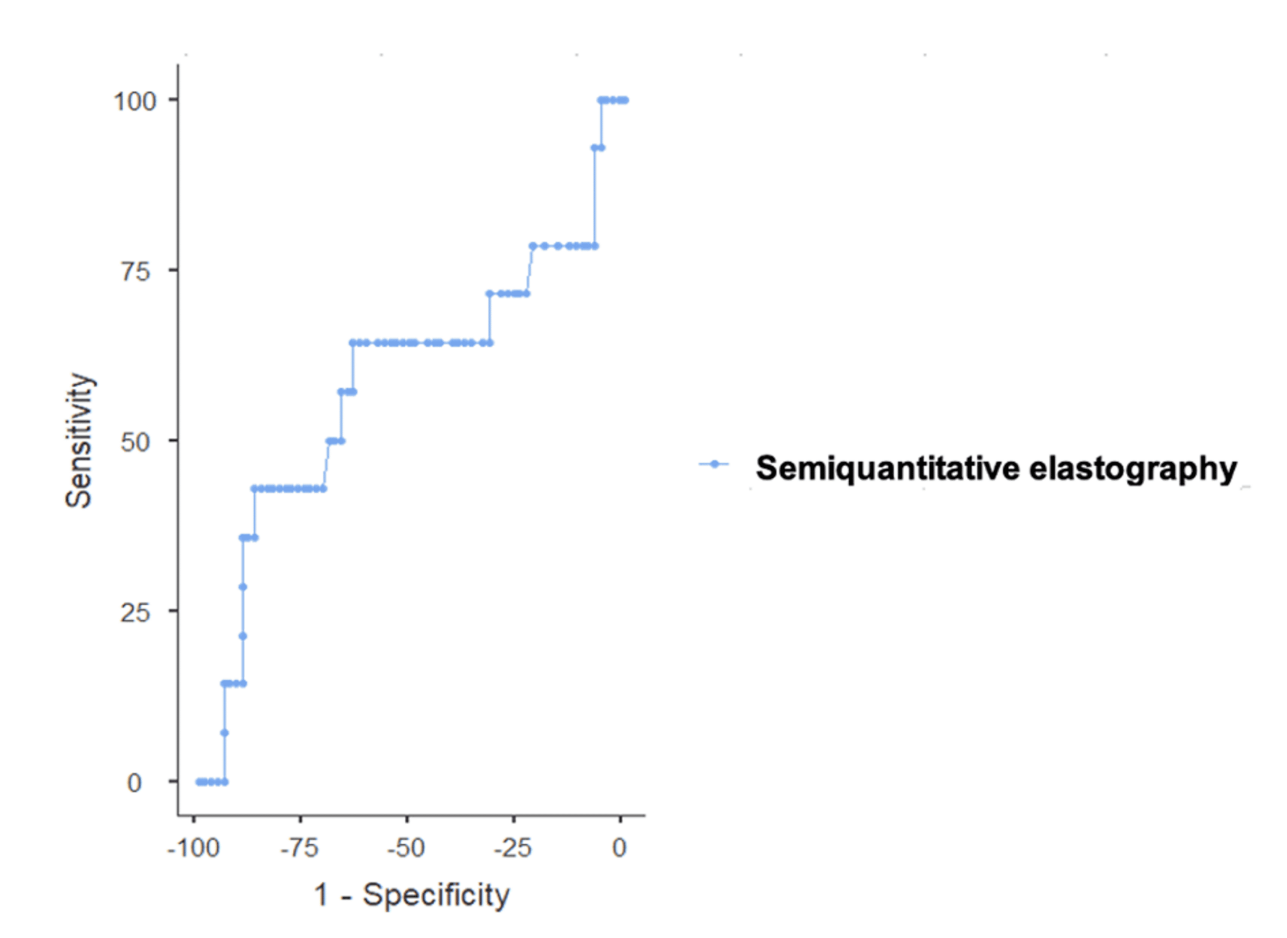
ROC curve, combined. Area under the ROC curve of 0.586 (95% CI: 0.40-0.78). ROC: receiver operating characteristic.

When exploring possible semiquantitative cutoff values for determining malignancy, an area under the ROC curve of 0.586 (95% CI: 0.40-0.78) was obtained (Figure 4). Two candidates are highlighted in the table: the cutoff of 1.48 provided the best balance between sensitivity of 64.29% (95% CI: 38.76-86.66) and specificity of 63.77% (51.98-74.1). The cutoff of 3.41 is also a strong candidate given its specificity of 86.96% (77.03-92.98), albeit at the cost of reduced sensitivity (42.86%) (Table 4).

**Table 4:** Diagnostic performance of semiquantitative elastography for determining malignancy in thyroid nodules (malignant vs. benign). PPV: positive predictive value; NPV: negative predictive value. Analysis performed on 83 nodules (14 malignant and 69 benign).

Cutoff value	Sensitivity (%)	Specificity (%)	PPV (%)	NPV (%)	Youden Index
1.41	64.29	60.87	25.00	89.36	25.16
1.45	64.29	62.32	25.71	89.58	26.61
1.48	64.29	63.77	26.47	89.80	28.06
3.01	42.86	82.61	33.33	87.69	25.47
3.08	42.86	84.06	35.29	87.88	26.92
3.38	42.86	85.51	37.50	88.06	28.37
3.41	42.86	86.96	40.00	88.24	29.82
3.92	35.71	89.86	41.67	87.32	25.57

## Discussion

In the context of the growing global detection of thyroid nodules and thyroid cancer, the present study is the first in Colombia with a reasonable sample size to evaluate the performance of elastography, used alongside routine ultrasound assessment of thyroid nodules, in comparison with the gold standard for malignancy diagnosis, i.e., cytopathology. The results demonstrated low concordance between elastography and cytopathology, underscoring the need for further research addressing interobserver analysis, different elastography modalities, and the use of the technique on various equipment platforms.

In this study, consistent with the literature, thyroid nodules were found to be more prevalent in women, and most patients had no prior history of thyroid malignancy [14,15]. The ACR TI-RADS classification system was used at the time of biopsy, showing good concordance between the referral classification and that assigned at the time of the procedure, with TI-RADS 4 nodules being the most prevalent. Nodule cytopathology was classified by the Bethesda System, identifying that the majority of thyroid nodules in the study population were benign (n = 69, 83.1%), with malignancy detected in only 14 patients (16.9%), consistent with the global literature, where more than 80% of nodules are benign and fewer than 10% are malignant [4,9,14].

In analyzing the application of elastography, the present study employed both semiquantitative and qualitative scoring systems. The semiquantitative values were similar across all cytopathology categories, indicating limited utility in discriminating between benign and malignant nodules in this population. Nevertheless, malignant nodules classified as Bethesda IV and V had mean qualitative values above 2. The literature has examined various scales, including the A score for qualitative assessment, with multiple studies reporting differing cutoff values for determining malignancy [6,7,12]. Kura et al. (2014) established a cutoff of ≤2 with a 99% negative predictive value (NPV) for benignity [15]. Mena et al. suggest a cutoff of 2.69, with a sensitivity of 84% and a specificity of 57% [6]. Yoo et al. explain that variability in cutoff values stems from the heterogeneous nature of nodule images, which leads operators to select a region of interest (ROI) that differs depending on the patient, the degree of nodule fibrosis, and the histological tumor subtype [16], making it difficult to establish a consistent and precise threshold. Hahn et al. (2018) identified 1.71 as the optimal cutoff [17]. In 2017, the World Federation for Ultrasound in Medicine and Biology recommended a standardized cutoff of 3.79, with a sensitivity of 97.8% and a specificity of 85.7%; however, that dataset showed considerable heterogeneity, with values ranging from 1.5 to above 5 [18].

In the present study, a cutoff value of 1.48 demonstrated a reasonable balance between sensitivity (64.29%) and specificity (63.77%), but is particularly notable for its NPV of 89.8%, making it a strong candidate for ruling out malignancy, as are the other cutoff values analyzed below this threshold (Table 4). The cutoff of 3.41 offers a good NPV of 88.24% with a high specificity of 86.96%, but at the cost of reduced sensitivity (42.86%).

Concordance between qualitative elastography and cytopathology was low in the present study: 48 nodules (69.5%) classified as benign (Bethesda II) had A score values below 2, while only nine (24.3%) of malignant nodules had an A score of 3 or 4. These findings align with the current literature, which reports similar limitations. Nell et al. suggest that biopsy may be omitted in patients with an A score of 1, given its 99% NPV, and report a good Kappa index of 0.64 for qualitative analysis; however, in their own systematic review’s interobserver analysis, concordance was low with a Kappa of 0.5 [11].

The main limitations of the present study relate primarily to the qualitative assessment of nodules, which, like conventional ultrasound, is operator-dependent and varies according to the radiologist assigning the score for each nodule, making the technique difficult to reproduce. Another potential limitation was the semiquantitative measurement approach, as the placement of the ROI by the radiologist may vary between the nodule itself and the surrounding healthy tissue. In our study, semiquantitative elastography (strain elastography) was used, which is more operator-dependent and has greater interobserver variability, unlike the quantitative technique (shear-wave elastography), which has less interobserver variability and is less operator-dependent [16].

Differences among elastography subtypes (qualitative, semiquantitative, and quantitative) remain the subject of ongoing debate in the literature. Even when using the semiquantitative method, false positives can occur when healthy thyroid tissue is inadvertently included in the measurement area, as demonstrated in several studies [19-22]. Other studies have shown that the semiquantitative method performs equivalently to the quantitative approach [23,24]; in contrast to Cantisani et al. (2019), who reported superior performance of the semiquantitative over the quantitative method [7]. Based on this evidence, the present study employed the semiquantitative method, which was the approach used during the radiologist training at the medical center.

The application of elastography is subject to equipment-related technical factors: the devices used in this study were of the same brand and utilized identical software, and no data were available for correlation with other brands or software platforms using different elastography approaches. Patient-related factors, such as neck positioning or hyperextension, carotid pulsatility, and respiration, may also influence the pressure applied by the radiologist over the nodule, and no interobserver calibration was performed for these variables.

Our study has an acceptable sample size with an applied clinical design appropriate to the study type. Although significant concordance was not achieved and sensitivity and specificity were not particularly high, numerous studies have demonstrated excellent sensitivity and specificity of elastography for distinguishing malignant from benign nodules. This highlights the need for additional studies in Colombia using more modern equipment and software, employing the quantitative technique to reduce interobserver variability and reduce operator dependence. Adequate training of all radiologists based on the specific equipment available at their institution, with validation of intra- and interobserver variability, is essential.

The results of this study demonstrate low concordance between qualitative elastography values and the malignant or benign nature of thyroid nodules, and semiquantitative values likewise do not allow for the establishment of a precise numerical cutoff to define malignancy. For this reason, cytopathologic evaluation of all thyroid nodules suspicious for malignancy should continue to be the standard of care in clinical practice. Elastography has not demonstrated sufficient utility in this study to replace invasive procedures or to allow more precise selection of patients who should undergo biopsy, given the clinical significance of thyroid cancer in this setting.

## Conclusions

Thyroid nodules are a common finding on thyroid ultrasound. Elastography is a noninvasive ultrasound-based technique that provides qualitative and semiquantitative information about these nodules. In this study, thyroid elastography demonstrated low concordance with cytopathology, the gold standard for diagnosing thyroid cancer, with limitations in both qualitative and semiquantitative interpretation. Although it remains a promising tool, studies with improved technical standardization and larger sample sizes are needed to define its diagnostic utility in clinical practice and to continue advancing noninvasive approaches for the early detection of thyroid cancer.
